# Herbal toothpaste craze: A marketing triumph or a public health concern

**DOI:** 10.6026/973206300214543

**Published:** 2025-12-15

**Authors:** Mohit Kumar, Neha Chitkara, Vivek Vardhan Gupta, Soni Tuteja, Karan Shahi, Gaurav Garg, Bhagyesh Ohri

**Affiliations:** 1 Department of Community Medicine, Christian Medical College, Ludhiana, Punjab, India; 2 Department of Oral Medicine & Radiology, Christian Dental College, Ludhiana, Punjab, India; 3 Department of Public Health Dentistry, Christian Dental College, Ludhiana, Punjab, India; 4 Department of Periodontics & Oral Implantology, Desh Bhagat Dental College & Hospital, Mandi Gobindgarh, Punjab, India; 5 Intern, Christian Dental College, Ludhiana, Punjab, India

**Keywords:** Herbal, toothpaste, marketing, oral health, hygiene

## Abstract

Despite the growing popularity of herbal toothpastes, there is limited awareness among consumers regarding their actual composition, potential risks or the importance of fluoride in caries prevention. This cross-sectional survey among 300 respondents of rural centres of a medical college found that 68% used herbal toothpaste with major influence through television adverts. Although 72% were convinced that herbal toothpastes are free from chemicals, only 18% knew of hazards like enamel wear and awareness of fluoride was comparatively low. Most participants complained of dental problems without attributing them to toothpaste use, although 83% indicated a willingness to revisit their decision after receiving information and 72% would like to be advised by dental practitioners. Consumers are open to professional advice, highlighting an urgent need for public health education and stricter labeling.

## Background:

Oral healthcare has a rich and long history, with the first recorded toothpaste preparations in ancient China and India as early as 300-500 BC [1]. Good oral health is most critical for the prevention of diseases that are multifactorial in nature, such as dental caries and periodontal diseases, both primarily caused by dental plaque. It involves the proper use of a variety of oral hygiene items, including toothbrushes, mouthwashes and toothpaste [2]. In the recent past, there has been a considerable shift in the trend of consumer demand, particularly in India, from the conventional allopathic medicines towards local medicine practice and herbal formulations [3]. This is reflected in the global market for herbal toothpaste, which was at an estimated 1.5 billion USD in the year 2018 [4]. India is a significant player in this marketplace and, with a high percentage of 21.0% of total purchases made throughout the Asia-Pacific region [5], has been driving growth for the sales of the wide variety of herbal toothpastes entering the Indian market [6]. These products are generally sold and marketed as a chemical-free, safer version of the regular fluoride-based toothpaste and Indian marketing in rural India employs natural themes and celebrity endorsements to associate the product with a higher image [7, 8]. However, the widespread use of herbal toothpastes is generally motivated by the misconception that "natural" translates to "chemical-free" and inherently safer [9, 10]. Both herbal and commercial toothpastes contain active and inactive components essential to their efficacy and stability. While herbal toothpastes utilize natural abrasives like charcoal, salt and neem, exaggerated abrasivity leads to dentine hypersensitivity and loss of enamel [9, 10]. Of equal concern is the use by most herbal toothpastes of either little or no fluoride, a long-standing agent essential in caries prevention [11, 12]. Misinformation about fluoride, even after massive studies affirming its benefits, has created skepticism among rural populations with minimal access to professional dental advice [13, 14]. In addition to issues of composition, regulatory issues complicate the landscape of herbal toothpastes as well. In contrast to standard toothpastes, which undergo rigorous quality control procedures, some herbal products lack standardized testing and therefore yield mixed concentrations of ingredients and variable levels of efficiency [15]. Label variation in the marketing of herbal products has also been noted by studies, creating problems for consumer clarity and consistency of product [16]. There has also been research that has shown different toothpastes, even natural toothpastes, have been reported to affect the surface characteristics of teeth and restorations [17, 18-19]. This trend reflects the necessity for increased public education and regulation. While herbal ingredients hold promise, the current trend towards relying more on marketing messages than on scientific reality carries with it inherent risks to oral health. Health awareness campaigns combined with evidence-based communications are required to allow the consumer to make the right choice rather than making decisions solely based on marketing factors [20]. On this premise, this study aims to formulate poly-herbal toothpaste and investigate its antimicrobial activity. Therefore, it is of interest to investigate the factors influencing the adoption of herbal toothpastes in rural populations, assessing consumer knowledge and perceptions and highlighting associated oral health implications.

## Methodology:

A cross-sectional survey was conducted among 300 individuals from rural areas of Ludhiana, Punjab, to assess the preferential shift from non-herbal to herbal toothpastes driven by television advertisements and the belief that herbal toothpastes are completely chemical-free. The study aimed to evaluate consumer awareness regarding the composition and potential adverse effects of herbal toothpastes, such as enamel abrasion and lack of fluoride. Participants were recruited by a convenience sampling technique from the rural centres of Christian Dental College, Ludhiana. Adults aged 18 years and older who used toothpaste daily were included, whereas participants with professional dental education, including dentists and dental students, were excluded. A standardized questionnaire was prepared to obtain information regarding demographic characteristics, preferences for toothpaste, reasons behind using herbal or non-herbal toothpastes, knowledge about their chemical constituents and awareness of possible side effects. The questionnaire was tested with a pilot study on 10 subjects and adjustments were made as and when required for clarity. The data were collected via face-to-face interviews to maintain precision and accuracy of response. The main variables investigated were the percentage of participants that utilized herbal and non-herbal toothpastes, why they chose them, the use of television advertisement as a source of influence for product choice and knowledge about toothpaste composition and impacts. Informed consent was obtained from all the participants prior to data collection. Confidentiality of the responses was ensured and participation was voluntary. Descriptive statistics were employed to present the data and the associations of toothpaste preference with awareness levels were examined using the chi-square test. A p-value of less than 0.05 was regarded as statistically significant.

## Results:

Among 300 participants, 165 (55%) were male and 135 (45%) were female. The majority of respondents (67%) had an education level below the secondary level and 80% belonged to a lower socioeconomic background (Table 1 - see PDF). A majority of participants (68%) reported using herbal toothpaste, while 32% continued using non-herbal varieties. The most common reason for switching to herbal toothpaste was television advertisements (79%), followed by perceived health benefits (53%) and the belief that herbal toothpaste contains no harmful chemicals (47%) (Table 2 - see PDF). A substantial proportion (72%) believed that herbal toothpaste was completely chemical-free and 64% assumed it was safer than non-herbal alternatives. However, only 18% were aware of potential adverse effects such as enamel erosion. Additionally, 57% of participants were unaware of whether their toothpaste contained fluoride (Table 3 - see PDF). When asked about their oral health experiences after switching to herbal toothpaste, 41% of herbal toothpaste users reported symptoms of dental sensitivity or increased tooth wear. However, they did not attribute these issues to their toothpaste choice (Table 4 - see PDF). After being informed about the potential risks associated with herbal toothpaste, 83% of participants stated they would reconsider their toothpaste choice and preferred to receive more information from dental professionals (Table 5 - see PDF).

## Discussion:

The present study identifies a remarkable change in toothpaste use among Ludhiana rural population, dominated by television advertisements and widespread misinformation about herbal toothpaste. Our research revealed that 68% of the participants used herbal toothpaste, with 79% of them ascribing it to television advertisements as the main influence. This is consistent with previous studies by Logaranjani *et al.* which demonstrated how advertising campaigns, particularly those highlighting herbal products as chemical-free and natural, deeply influence people in developing nations [7]. The general perception that herbal toothpaste is "better for health" (53%) and "no dangerous chemicals" (47%) supports this trend quite frequently irrespective of the sanction of science. A disturbing finding in our research was the overall lack of knowledge of the constituents and potential side effects of herbal toothpaste. We found that 72% of the subjects perceived herbal toothpaste to be completely free from chemicals and 64% perceived it to be safer than conventional toothpastes. Similar misinformation has been documented in previous studies. Kumar *et al.* and Athawale *et al.* for instance, found that customers are prone to interpret "herbal" as "harmless," so they disregard abrasivity and fluoride depletion issues [9, 10]. Our research revealed that 18% of consumers of herbal toothpaste were aware of the risk of enamel erosion, significantly less than non-herbal toothpaste customers (49%) (p<0.05). This concurs with existing studies that have proven consumers will tend to underestimate the contribution of abrasives in toothpaste composition and resulting in long-term dentine wear [11, 12]. The absence of fluoride in herbal toothpaste is a serious matter. In this study, 57% of the respondents were unsure if their toothpaste contained fluoride, while a mere 18% of users of herbal toothpaste were aware of its role in dental caries prevention. This finding is consistent with the findings by Prasad *et al.* and Lotto *et al.* whose findings showed that the lack of fluoride in herbal toothpaste could enhance dental caries susceptibility, particularly among those with limited access to professional dental care [13, 14]. The safety myth of fluoride has been maintained through advertisement and media misinformation, which lowered the acceptance of fluoride products, as reported by Balekundri & Mannur [15]. One of our concerns in the findings was that 41% of the patients of toothpaste with herbal active ingredients experienced dental sensitivity or increased tooth wear after switching but did not link these to their toothpaste. This absence of correlation is supported by research conducted by Boloor *et al.* and Bashirian *et al.* which explains that patients will accuse tooth troubles of being caused by something else, such as diet or brushing action, before accusing their toothpaste [16, 20]. Encouragingly, when informed about the possible risks associated with herbal toothpaste, 83% of our sample was willing to change their minds and 72% wished to be informed by experts. This implies that well-targeted education interventions had the potential to make a large impact on consumer education and oral health outcomes. These kinds of programs have worked in other places; Hashmi *et al.* and Qin *et al.* demonstrated that expert advice can effectively reverse incorrect understanding and establish improved oral hygiene habits [21, 22]. The popularity and popularity of herbal toothpastes currently on supermarket shelves demonstrate the need to ascertain if such products can cause changes in surface properties of aesthetic restorative materials. This was explored in a study by Hazra *et al.* which emphasized that healthcare professionals need to be sensitized to such limitations and inform the patients of them while providing aesthetic restorations [23]. Kumar *et al.* concluded that nearly 75% of the respondents in their research had a preference for herbal toothpastes over non-herbal toothpastes and 44.8% attributed their preference by stating that herbal toothpastes are harmless and do not contain side effects or chemicals in them [24]. This problem of side effects and toxicity appears to be one of the major reasons for the observed trend towards herbal commodities, as this is also supported by Kumar *et al.* [25]. Second, Kumar *et al.* noted that while patients do switch toothpastes in a bid to obtain some relief from chronic problems like sensitivity of teeth and yellow teeth, the switching was not all of a sudden followed by better symptoms. Despite this, the majority of the patients still preferred herbal toothpastes since they thought they were "chemical-free" [25].

## Conclusion:

Major findings include public lack of knowledge regarding ingredients of toothpaste, the positive effects of fluoride and abrasive dangers of certain herbal products. In spite of this, the population is receptive to professional advice. These results emphasize the need for immediate public health education programs to correct misconceptions as well as the enforcement of stronger labeling by regulatory agencies.
